# Stability of small non-coding RNA reference gene expression in the rat retina during exposure to cyclic hyperoxia

**Published:** 2013-02-25

**Authors:** Melinda Tea, Michael Zenon Michael, Helen Mary Brereton, Keryn Anne Williams

**Affiliations:** 1 Department of Ophthalmology, Flinders University of South Australia, Adelaide, Australia; 2 Department of Gastroenterology and Hepatology, Flinders University of South Australia, Adelaide, Australia

## Abstract

**Purpose:**

Oxygen-induced retinopathy (OIR) is a robust animal model of human retinopathy of prematurity that readily allows changes in retinal gene and microRNA (miRNA) expression in response to fluctuations in oxygen levels to be studied. We sought to identify small non-coding RNA (ncRNA) genes that showed stable expression upon exposure to varying levels of oxygen, with different developmental stages and in different rat strains, to act as reference genes for normalizing miRNA expression in a rat model of OIR.

**Methods:**

Expression of five small ncRNAs (U6 snRNA, miR-16, U87, 4.5S RNA (H) “Variant 1”, and 5S ribosomal RNA [rRNA]) were tested on a standard RNA pool and representative retinal samples from P5, P6, P9, and P14 from room air– and cyclic hyperoxia–exposed rats using reverse transcription (RT)-qPCR, to assess the effect of developmental stage and exposure to fluctuations in oxygen levels, respectively. Two strains of inbred albino rats, Fischer 344 (F344, resistant to OIR) and Sprague-Dawley rats (SD, susceptible to OIR), were used to assess the effect of rat strain on the stability of the small ncRNAs.

**Results:**

In this rat model of OIR, 5S rRNA expression was variable with strain, fluctuations in oxygen levels, and developmental stage. U6 snRNA was stably expressed with changes in oxygen levels, and minimal variation was observed with strain and developmental stage. MiR-16 showed less stable expression with changes in oxygen levels and between strains compared to U6 snRNA. Some variation in expression in response to developmental stage was also observed. The PCR amplification efficiencies of the U6 snRNA and miR-16 TaqMan assays were 56% and 78%, respectively. U87 and 4.5S RNA (H) “Variant 1” expression varied with strain, exposure to cyclic hyperoxia, and in particular developmental stage, and was at low levels in the neonatal rat retina.

**Conclusions:**

We conclude that U6 snRNA and miR-16 are the most suitable reference RNAs for normalizing miRNA expression, as they are relatively stable with strain, exposure to cyclic hyperoxia, and developmental stage in a rat model of OIR.

## Introduction

Normalization of reverse transcription (RT)-qPCR data using stably expressed reference genes is essential for accurately profiling gene and microRNA (miRNA) expression [1-4]. Genes that are constitutively expressed and are associated with housekeeping or structural functions are commonly used as reference genes, as they tend to be expressed across many cell and tissue types and show minimal variation among samples or experimental conditions [4-7]. However, evidence suggests that the expression of commonly used reference genes such as glyceraldehyde 3-phosphate dehydrogenase (GAPDH) and beta-actin varies between cell and tissue types [8,9]. Identifying and characterizing stably expressed reference genes through a series of preliminary experiments is recommended, to accurately determine gene and miRNA expression levels and to avoid incorrect conclusions being drawn [3,6,10,11].

Retinopathy of prematurity (ROP) is a potentially blinding condition that affects premature infants who undergo supplemental inspired oxygen therapy. Oxygen-induced retinopathy (OIR) is a robust animal model of ROP that mimics the pathology seen in human disease [12,13]. The rat model of OIR described in this study utilizes exposure of neonatal rats to cyclic hyperoxia and relative hypoxia [14-17]. As changes in retinal gene expression in response to exposure of neonates to hyperoxia and relative hypoxia are involved in inducing OIR [18-21], identifying references genes that are stable with variations in oxygen levels is vital. Previous studies from our laboratory identified two RNA reference genes encoding acidic ribosomal phosphoprotein and hypoxanthine guanine phosphoribosyl transferase as being stably expressed in response to cyclic hyperoxia and during early retinal development in several different rat strains [5], making these reference genes suitable for normalizing gene expression data.

MicroRNAs expressed in the eye are associated with physiologic and pathological processes and display tissue specificity and spatiotemporal expression [22,23]. In mouse models of OIR, miRNAs regulate retinal angiogenesis by post-transcriptional modification of genes involved in the angiogenic response to hypoxia [24,25].

5S ribosomal RNA (rRNA), a commonly used reference transcript for miRNA RT-qPCR, has been used to normalize miRNA expression in mouse models of OIR [24-26]. Unlike the reference genes used in normalizing gene expression that are typically protein-coding genes, the reference genes used for normalizing miRNA expression should, in general, be small non-coding RNAs (ncRNAs) such as small nuclear and small nucleolar RNAs, given that the total RNA isolation process biases toward the recovery of large RNAs over small ncRNAs [4,27]. However, the choice of the correct normalization RNA appears to depend on the context in which miRNA expression is being investigated [10,28]. Whether 5S rRNA is the most appropriate small ncRNA for normalizing miRNA expression in rodent models of OIR is currently unknown, as stable expression of 5S rRNA with changes in oxygen levels has not previously been established. We sought to identify small ncRNAs that showed stable expression upon exposure to varying levels of oxygen, in different rat strains and at different developmental stages to act as reference genes for normalizing miRNA expression in a rat model of OIR.

## Methods

### Experimental animals

Albino inbred Fischer 344 (F344; resistant to OIR) and Sprague-Dawley (SD; susceptible to OIR) rats and pigmented inbred Dark Agouti (DA; susceptible to OIR) rats were obtained from the institutional animal facility. Rats were allowed unlimited access to rat chow and water and were exposed to a 12 h:12 h light-dark cycle. Temperature was maintained at 24 °C in a humidified atmosphere. Animals were euthanized with an inhaled overdose of isoflurane anesthetic. All experiments were approved by the institutional Animal Welfare Committee and met the standards described in the Association for Research in Vision and Ophthalmology Statement for the Use of Animals in Ophthalmic and Vision Research and those set out by the Australian Code of Practice for the Care and Use of Animals for Scientific Purposes.

### Exposure of neonatal rats to cyclic hyperoxia

The oxygen exposure protocol used in this study was a modification of those previously used in other studies of OIR in the rat [15-17,29,30]. Within 12 h of birth, female rats and their litters were placed in a custom-built humidified chamber designed to deliver oxygen to neonatal rat litters under controlled conditions. Rats were exposed to alternating 24 h cycles of hyperoxia (80% oxygen in air) and room air (normoxia; 21% oxygen in air) for a maximum of 14 days after birth. Age- and strain-matched room air–exposed rats were used as controls.

### Preparation of total RNA from rat retinas

Total RNA was prepared from the retinas of neonatal rats as described previously [31], using TRIzol (Invitrogen, Carlsbad, CA) according to the manufacturer’s method. Total RNA was also prepared from the rat liver, brain and lung, and mouse lens and liver using the same method. Mouse liver and lens tissue were kindly provided by Dr. Shiwani Sharma (Flinders University, Adelaide, South Australia). Quantification of total RNA was performed using the NanoDrop 8000 (Thermo Scientific, Wilmington, DE). An absorbance ratio of the RNA at 260 and 280 nm was used as a guide to determine RNA purity. Total RNA with a ratio of 1.8 or more was considered acceptable. Agarose gel electrophoresis was used to assess RNA integrity and residual DNA contamination. Total RNA with a 28S:18S ratio of approximately 2:1 and showing minimal DNA contamination was considered acceptable for use.

### Preparation of cDNA for qPCR

cDNA was prepared from total RNA for each individual sample from room air- and cyclic hyperoxia-exposed F344 and SD rats at P5, P6, P9, and P14 using a method described previously [31]. Briefly, one microgram of total RNA was DNase-treated with Turbo DNAfree (Ambion, Austin, TX) according to the manufacturer’s instructions and reverse-transcribed in 20 μl reactions using a first-strand cDNA synthesis kit (SuperScript III First-Strand Synthesis System, Invitrogen) and random hexamer primers. An RT-free control sample was prepared in parallel with each cDNA sample, in which diethylpyrocarbonate (DEPC)-treated water was used in place of reverse transcriptase. Relative quantification of gene expression was performed on cDNA from each individual sample at a 1:5 dilution. A single representative room air– or cyclic hyperoxia–exposed rat was used at each time point for each strain of interest.

cDNA was also prepared from a standard RNA sample made from retinal RNA pooled from several room air– and cyclic hyperoxia–exposed albino F344 and SD rats and pigmented DA rats from various developmental stages, ranging from P2 to P14. Each sample was represented equally in terms of RNA yield. The standard pool was prepared to allow for normalizing RT-qPCR data for gene and small ncRNA expression in the context of OIR. The standard pool was prepared to allow all strains, treatment groups, and developmental stages to be covered, as well as providing sufficient RNA for normalization across multiple experiments to be performed.

### RT-qPCR analysis of 5S RNA expression

RT-qPCR was performed with reference to the Minimum Information for Publication of Quantitative Real-Time PCR Experiments guidelines [32]. Each 10 μl reaction mixture contained 5 μl of Power SYBR Green PCR Master Mix (Applied Biosystems, Foster City, CA), 1 μl each forward and reverse primers (0.5 μM final concentration), and 3 μl cDNA sample diluted 1:5 with Ultrapure water (Fisher Biotech, WA, Australia). Reactions were run on a StepOne Plus real-time PCR System (Applied Biosystems). The cycling conditions were initial denaturation (95 °C, 15 min) followed by 40 cycles of denaturation (95 °C, 15 s), annealing, and extension (60 °C, 30 s).

Primers specific for rat 5S rRNA were published previously [33]. The sequences for each primer were as follows: The forward primer was 5′-TCT CGT CTG ATC TCG GAA GC-3′, and the reverse primer was 5′-AGC CTA CAG CAC CCG GTA TT-3′. Primers were synthesized by Geneworks Ltd (Thebarton, South Australia, Australia).

### Assays for RT-qPCR analysis of miRNA expression

TaqMan miRNA assays (Applied Biosystems) were used to determine miRNA expression levels in the retinal, liver, brain, lung, and lens samples. Eight commercially available small ncRNA controls, referred to as “endogenous controls” by Applied Biosystems, were chosen for analysis. In addition to these endogenous controls, four miRNAs considered stably expressed with strain and treatment were included. They were manually identified from data derived from Exiqon miRNA arrays (Exiqon A/S, Vedbaek, Denmark) performed at the Adelaide Microarray Centre (Adelaide, Australia) using Exiqon miRNA version 8.1 and version 11 all species array libraries. Briefly, microarray libraries were printed onto slides. Retinal-derived total RNA were labelled, hybridized and scanned according to the manufacturer’s protocol. Preliminary Exiqon microarray data analyses were performed at the Adelaide Microarray Centre using the software package LIMMA R (WEHI, Melbourne, VIC, Australia) to identify differentially expressed miRNAs. The full microarray processing protocol and statistical analyses has been described elsewhere [34]. The arrays were used to identify miRNAs that were differentially expressed as a result of rat strain and/or changes to oxygen levels. Thirteen ncRNAs (including 5S rRNA) were tested before RT-qPCR quantification of miRNA expression. The corresponding Applied Biosystems TaqMan miRNA assay reference numbers are shown in Table 1. Primer amplification efficiencies for each small ncRNA were determined using the standard RNA sample.

**Table 1:** Small ncRNA suitability as reference genes for normalization of miRNA expression in rat retinal samples.

**Small ncRNA**	**Species reactivity**	**Assay ID**	**Amplification efficiency in rat retina**	**Order of magnitude**	**R^2^ value**	**Mean Ct**
4.5S RNA(H)* “Variant 1”	Rat	001716	49.3%	2.4	1.00	24.1
4.5S RNA(H)* “Variant 5”	Rat	001717	ND – low abundance	ND	ND	ND
snoRNA202*	Mouse	001232	ND – low abundance	ND	ND	ND
snoRNA*	Rat	001718	ND – low abundance	ND	ND	ND
U6 snRNA*	Human, mouse and rat	001973	56.2%	4.2	1.00	20.9
RNU6B*	Human	001093	ND – low abundance	ND	ND	ND
Y1*	Rat	001727	ND – low abundance	ND	ND	ND
U87*	Rat	001712	62.0%	2.4	0.98	23.8
5S rRNA	Rat	N/A	92.5%	4.0	0.98	23.0
hsa-miR-16 ^+^	Human, mouse and rat	000391	77.5%	4.2	0.98	24.2
mmu-miR-379^+^	Human, mouse and rat	001138	ND – low abundance	ND	ND	ND
hsa-miR-191^+^	Human, mouse and rat	002299	ND – low abundance	ND	ND	ND
hsa-let-7d^+^	Human, mouse and rat	002283	ND – low abundance	ND	ND	ND

Thirteen small ncRNAs were tested for miRNA normalization. Applied Biosystems assay ID numbers are provided for all small ncRNAs other than 5S rRNA, which was not available as an Applied Biosystems TaqMan assay. PCR primer amplification efficiencies were determined using serial dilutions of the standard RNA pool to a minimum of 2 orders of magnitude to generate a standard curve. The order of magnitude of the standard curve dilution series, the R^2^ value for each standard curve and the mean Ct across all test samples, including the standard pool is shown. N/A=not available; ND=not able to be determined; *=small ncRNA assays referred to as endogenous controls by Applied Biosystems; +=miRNA identified from in-house Exiqon microarrays as being stably expressed with strain and oxygen exposure.

### RT-qPCR analysis of miRNA expression

cDNA synthesis and qPCR reactions were performed using a StepOne Plus real-time PCR System (Applied Biosystems). Conversion of total RNA to single-stranded cDNA was performed in accordance with the manufacturer’s instructions for the TaqMan miRNA assays with slight modifications as detailed. Briefly, total RNA samples were diluted to a concentration of 4, 20, or 40 ng/μl depending on the optimized template concentration determined for each miRNA of interest. A total of 5–40 ng of RNA was used in each reverse transcription reaction.

The reverse transcription reaction was modified to 7.5 μl rather than 15 μl reactions and the volumes adjusted accordingly. A bulk reverse transcription master mix was prepared containing 100 mM deoxynucleotide triphosphates (with deoxythymidine triphosphate, 0.075 μl/reaction), Multiscribe™ Reverse Transcriptase 50 U/μl (0.5 μl/reaction), 10 x Reverse Transcriptase Buffer (0.75 μl/reaction), RNase Inhibitor 20 U/μl (0.095 μl/reaction) and nuclease-free water (2.08 μl/reactions). The reverse transcription master mix (3.5 μl/reaction) was then aliquoted into a 96 well reaction plate with 0.1 ml well volume (Applied Biosystems) and miRNA-specific RT primer (1.5 μl/reaction) added. The diluted total RNA (2.5 μl/reaction) was then added to bring the final volume to 7.5 μl.

The reactions were incubated for 30 min at 16 °C and then followed by 30 min of incubation at 42 °C. The reaction was then terminated at 85 °C for 5 min before being held at 4 °C until required for qPCR that was performed on the same day.

A PCR master mix was prepared for each reference small ncRNA gene. Each 10.3 μl reaction contained 0.5 μl 20X TaqMan miRNA assay mix, 5 μl TaqMan 2X Fast Universal PCR Master Mix No Amperase UNG, 3.8 μl of nuclease-free water, and 1 μl of the reverse transcription product. Each reaction was performed in triplicate. Nuclease-free water was used in place of the reverse transcription template as a negative control for non-specific amplification. PCR reactions were placed in the StepOne Plus cycler, and the AmpliTaq DNA polymerase was activated at 95 °C for 20 s, followed by 40 cycles of denaturing at 95 °C for 1 s, and then annealing and extension at 60 °C for 20 s. Again, a single representative room air– or cyclic hyperoxia–exposed rat was used at each time point for each strain of interest.

### Determination of PCR primer amplification efficiencies

PCR primer amplification efficiencies were determined for the 5S rRNA primer pair using ten threefold serial dilutions of the standard cDNA sample derived from rat retinal tissue to generate a standard curve. Dilutions were made over five orders of magnitude; however, reliable data were generated only to four logs. For miRNA primer pairs, nine fourfold serial dilutions of the standard RNA sample from rat retinal tissue were used to generate the standard curve. Depending on the miRNA primer pair, reliable data were generated to a minimum of two logs and a maximum of four logs. Although data from the standard curve would ideally extend to five orders of magnitude to determine PCR amplification efficiency accurately, the abundance of each mRNA or miRNA in the sample of interest was a limiting factor.

Standard curves for five small ncRNA primer pairs were also performed on total RNA derived from rat liver samples to determine if poor amplification efficiencies were due to the tissue-specificity of the ncRNA in question. In these instances, six twofold serial dilutions of the standard RNA sample were used to generate the standard curve over two orders of magnitude. A smaller dilution factor was used for the miRNA primer pairs to ensure that the majority of the threshold cycle values fell within the range of 20–30.

For all primer pairs, the mean threshold cycle value for each dilution was plotted against the log cDNA concentration, and the gradient of the regression line of the standard curve was used to calculate the PCR amplification efficiency. Amplification efficiencies were used in quantifying gene and miRNA expression in the context of OIR [35,36].

### Statistics

All statistical analyses were performed using the software package PASW Statistics version 18.0 (SPSS Inc., Chicago, IL). Independent-samples Student *t* tests or Mann–Whitney U tests were performed to compare stability of the small ncRNAs between strains or cyclic hyperoxia exposure, and the significance level (alpha) was set at 0.05. Statistical analysis for small ncRNA expression in response to development was performed using one-way between-groups analysis of variance (ANOVA), with the significance (alpha) level adjusted for multiple comparisons (Tukey’s honestly significant difference test) to p<0.0125. Where appropriate, a Kruskal–Wallis test with the significance (alpha) level set at 0.05 was used instead.

## Results

Eight small ncRNA endogenous controls available from Applied Biosystems, four miRNAs manually identified from in-house Exiqon microarray data to be stably expressed with strain and exposure to cyclic hyperoxia, and the small ncRNA 5S rRNA were tested using RT-qPCR. Primer PCR amplification efficiencies from total RNA derived from rat retinal samples were determined using the standard RNA sample (Table 1). The linearity of the primer pairs exhibited R^2^ values ranging from 0.98 to 1.00 over a minimum of two orders of magnitude. Amplification efficiencies were unable to be determined for some assays including 4.5S RNA (H) “Variant 5,” snoRNA202, snoRNA, and Y1. These small ncRNAs may not be highly expressed in the neonatal rat retina; therefore, expression was examined in the rat liver (Table 2) and the mouse lens and liver (data not shown). In rat liver, 4.5S RNA (H) “Variant 1” was expressed, however 4.5S RNA (H) “Variant 5” was not highly expressed and the PCR amplification efficiency could not be determined. Interestingly, the same expression pattern was observed in the rat brain and lung (data not shown), suggesting that the 4.5S RNA (H) variants are not expressed to the same extent in different rat tissues. Y1 expression levels were low in the rat retina and liver. No cross-reactivity of the mouse-specific snoRNA202 assay was observed in the rat retina or liver; however, expression was confirmed in the mouse lens and liver (data not shown).

**Table 2:** PCR primer amplification efficiencies of small ncRNA endogenous controls in rat liver.

**Small ncRNA**	**Amplification efficiency in rat liver**	**R^2^ value**	**Ct at 1/2 dilution**
4.5S RNA(H) “Variant 1”	44.6%	0.99	25.4
4.5S RNA(H) “Variant 5”	ND – low abundance	ND	33.1
snoRNA202	ND – low abundance	ND	33.0
snoRNA	50.4%	1.00	26.9
Y1	ND – low abundance	ND	34.5

Five small ncRNA endogenous controls were tested to determine the PCR primer amplification efficiencies using total RNA derived from rat liver samples. Six serial twofold dilutions of the total RNA were used to generate the standard curves for 4.5S RNA (H) “Variant 1” and snoRNA. Cts were unable to be generated for all six twofold serial dilutions for the remaining small ncRNAs; therefore amplification efficiencies for these assays could not be determined. The R^2^ value for each standard curve is shown, as is the mean Ct at a 1/2 dilution. ND=not able to be determined.

U6 snRNA, miR-16, U87, 4.5S RNA (H) “Variant 1”, and 5S rRNA were chosen for further analysis, as the expression levels of these small ncRNAs were adequate for determining primer PCR amplification efficiencies. The small ncRNAs’ stability with strain, treatment, and developmental stage was tested using representative samples from individual rats from P5, P6, P9, and P14, relative to the standard pool.

### Effect of strain

The expression of each small ncRNA was separated based on the rat strain. The small ncRNA reference gene commonly used for normalizing miRNA expression in OIR, 5S rRNA, was the least stable of the five tested (Table 3). Analysis of the average expression level of each small ncRNA, relative to the standard pool, confirmed that little variation was present in U6 snRNA, miR-16, U87, and 4.5S RNA (H) “Variant 1” expression. However, the changes in expression among strains were not statistically significant for any gene.

**Table 3:** Expression of five small ncRNAs and miRNAs relative to a standard pool tested for stability between rat strains.

**Assay**	**Expression relative to the standard pool in F344 rats**	**Expression relative to the standard pool in SD rats**	**Difference in mean expression between F344 and SD rats**	**p value**
U6 snRNA	0.48 (±0.13)	0.45 (±0.13)	0.03	0.70
miR-16	0.66 (±0.33)	0.56 (±0.23)	0.10	0.50
U87	0.93 (±0.19)	0.90 (±0.21)	0.03	0.56
4.5S RNA(H) “Variant 1”	0.96 (±0.08)	0.90 (±0.21)	0.06	0.47
5S rRNA	0.63 (±1.13)	0.47 (±0.39)	0.16	0.53

Average expression relative to the standard pool and the standard deviation for each strain is shown. F344 n=8, SD n=8.

### Effect of exposure to cyclic hyperoxia

Expression of each small ncRNA separated based on room air or cyclic hyperoxia exposure showed 5S rRNA was the least stable reference transcript; however, this was not statistically significant (Table 4). Expression of U87, miR-16, and 4.5S RNA (H) “Variant 1” was somewhat variable with exposure to cyclic hyperoxia, although not to the same extent as for 5S rRNA. U6 snRNA showed the smallest change in average expression with fluctuations in oxygen levels. Overall, no changes in response to cyclic hyperoxia exposure were statistically significant.

**Table 4:** Expression of five small ncRNAs and miRNAs relative to a standard pool tested for stability with exposure to cyclic hyperoxia.

**Assay**	**Expression relative to the standard pool in room air-exposed rats**	**Expression relative to the standard pool in cyclic hyperoxia-exposed rats**	**Difference in expression between room air- and cyclic hyperoxia-exposed rats**	**p value**
U6 snRNA	0.49 (±0.16)	0.47 (±0.11)	0.03	0.70
miR-16	0.67 (±0.26)	0.55 (±0.31)	0.12	0.41
U87	0.85 (±0.15)	0.98 (±0.22)	0.13	0.08
4.5S RNA(H) “Variant 1”	0.96 (±0.08)	0.90 (±0.21)	0.06	0.47
5S rRNA	0.28 (±0.25)	0.82 (±1.10)	0.54	0.20

Mean expression relative to the standard pool and the standard deviation are shown for each treatment. Room air-exposed rats n=8, cyclic hyperoxia-exposed rats n=8.

### Effect of developmental stage

**Figure 1:**
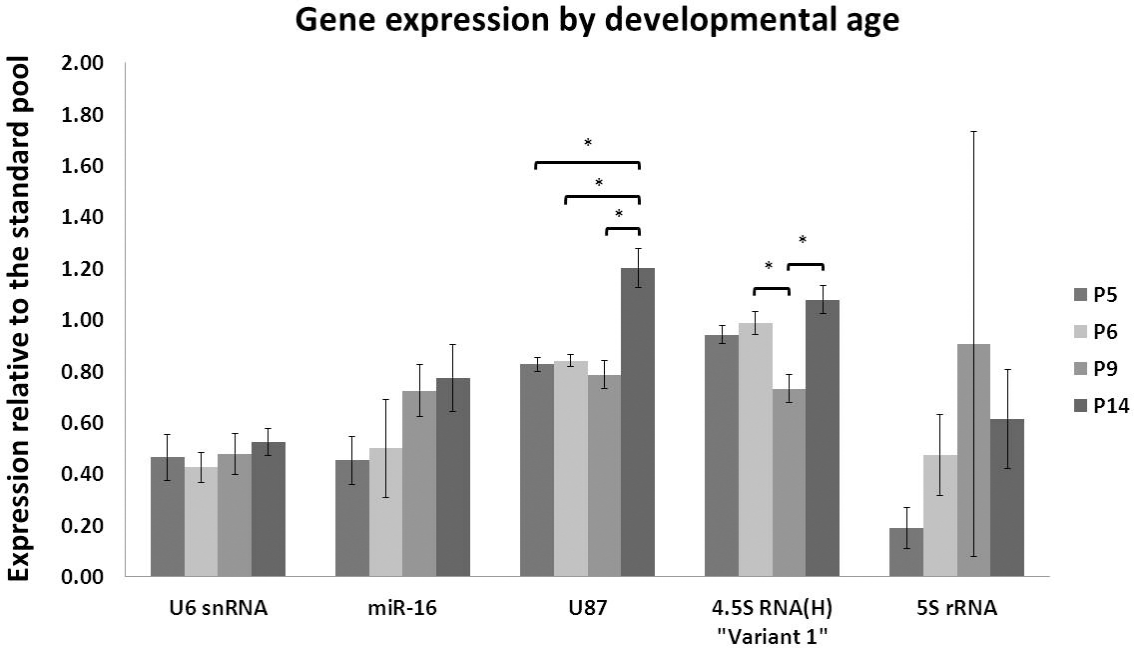
Average expression of five small ncRNAs and miRNAs compared at P5, P6, P9, and P14. Mean expression relative to the standard pool for each developmental stage. n=4 at each time point. Error bars represent ± standard deviation. Statistical analysis was performed using one-way ANOVA, with the significance level (alpha) adjusted for multiple comparisons (Tukey’s HSD test) at p<0.0125. For 5S rRNA, a Kruskal–Wallis test was used and the significance (alpha) level set at 0.05. * p<0.0125.

Levels of U6 snRNA and miR-16 showed slight increases with increasing developmental age; however, these differences were not statistically significant (Figure 1). U87 levels were stable between P5, P6, and P9; however, by P14, expression had increased to a statistically significant level after correction for multiple comparisons. Levels of 4.5S RNA (H) “Variant 1” varied with developmental stages. A statistically significant decrease in expression levels occurred between P6 and P9, and a statistically significant increase in expression levels was observed between P9 and P14. Levels of 5S rRNA, relative to the standard pool, increased with developmental age up to P9 and then decreased at P14.

## Discussion

The current convention for quantifying miRNA expression is to use a single small ncRNA reference gene for normalizing RT-qPCR data [37]. The choice of small ncRNA reference gene is important to avoid introducing bias or misleading conclusions. Gee et al. found that the expression of small ncRNA reference genes typically used for normalizing miRNA expression levels in cancer were themselves highly variable [10]. When these reference genes were used to normalize expression levels of miRNAs of interest from tumor samples, the potential prognostic value of these miRNAs was lost, as their normalized expression levels did not accurately reflect the associations between miRNA expression and tumor pathology or disease outcome [10]. These reference transcripts were later found to be derived from the introns of genes that themselves are dysregulated in cancer [10].

An ideal reference gene for use with rodent models of OIR is stable with strain, exposure to cyclic hyperoxia, and developmental stage. Previous studies of miRNA expression in mouse oxygen-induced retinopathy have used 5S rRNA as the reference RNA for normalizing miRNA expression [24-26], although the suitability of this reference gene for the purpose was hitherto unknown. Here, we showed that 5S rRNA levels varied with rat strain, changes in oxygen levels, and developmental stage, but this variability was not statistically significant. Some of the variation observed in 5S rRNA expression may be due to the different chemistries used to assay 5S rRNA expression levels (SYBR Green) compared with miRNA expression levels (TaqMan). However, expression of the 5S rRNA transcript was compared across samples within an assay method, rather than between methods, so that the variability is likely due to strain, oxygen exposure, or developmental stage. Previous studies of miRNA expression in mouse oxygen-induced retinopathy also used SYBR Green chemistry and TaqMan assays for analyzing 5S rRNA and miRNA expression levels, respectively [24,25]. Given that 5S rRNA expression levels varied between samples, we conclude that in at least this rat model of OIR, 5S rRNA is not a suitable reference RNA for normalizing miRNA expression levels.

U6 snRNA levels were stable with fluctuations in oxygen, strain, and developmental stage compared to the remaining four reference genes. However, the PCR amplification efficiency was only 56% for this particular assay. In comparison, miR-16 showed less stable average expression with changes in oxygen levels and between rat strains compared with U6 snRNA. Some variation in expression was observed with developmental stage, but less than that seen with 5S rRNA. The PCR amplification efficiency for the miR-16 TaqMan assay was better than for U6 snRNA, at 78%. The remaining two reference RNAs, 4.5S RNA (H) “Variant 1” and U87, showed poor PCR amplification efficiencies in neonatal rat retinas with efficiencies of 49% and 62%, respectively, and were variable with strain, exposure to cyclic hyperoxia, and developmental stage. Statistically significant changes in small ncRNA expression were observed in response to developmental stage for 4.5S RNA (H) “Variant 1” and U87.

5S rRNA has typically been used for normalizing miRNA expression levels in mouse models of oxygen-induced retinopathy, although the stability of this reference gene with changes in oxygen levels has not previously been examined. We have established that data derived from the use of 5S rRNA for normalization is statistically valid. However, the lower degree of variability observed in expression levels of U6 snRNA and miR-16 suggest they may be more appropriate for use in normalizing miRNA expression levels. Taken together, the data suggest that from the limited number of rat-reactive small ncRNA control TaqMan assays available U6 snRNA and miR-16 are most suitable for normalizing miRNA expression in rat models of OIR, as these reference genes are relatively stable with strain, exposure to cyclic hyperoxia, and developmental stage.
